# Return to Sport and Return to Self-Reported Preinjury Level After Surgical Repair of Acute Achilles Tendon Rupture: A 2-Year Prospective Cohort Analysis

**DOI:** 10.3390/jcm15176512

**Published:** 2026-08-23

**Authors:** Maxime Guerot, Frederic Khiami, Eugénie Valentin, Olivier Grimaud, Mohamad K. Moussa, Antoine Gerometta, Alain Meyer, Nicolas Lefèvre, Alexandre Hardy

**Affiliations:** Clinique du Sport, Groupe Chirurgie du Sport, 75005 Paris, Francealexandre.hardy@me.com (A.H.)

**Keywords:** Achilles tendon rupture, return to sport, sport surgery

## Abstract

**Background:** Achilles tendon ruptures are among the most common sports injuries. The literature reports highly variable “return to sport” (RTS) rates depending on the study, as the definition of return to play is not consistent. Our purpose was to assess RTS and self-reported relative performance level after surgical management of acute Achilles tendon ruptures. **Method:** In this retrospective analysis of a prospective cohort, the RTS rate and the return to self-reported preinjury level (RTPIL) in patients treated surgically for acute Achilles tendon rupture between November 2020 and September 2023 with at least 2 years of follow-up were reported. The primary outcomes were the RTS and RTPIL rates at last follow-up. Secondary outcomes were Patient-Reported Outcome Measures (PROMS): Achilles Tendon Total Rupture Score (ATRS), Ankle Ligament Reconstruction–Return to Sport after Injury (ALR-RSI), Foot and Ankle Ability Measure (FAAM), Tegner Activity Scale (TAS) and the University of California and Los Angeles (UCLA) activity level rating scale. Data was collected preoperatively; at three, six, twelve, and twenty-four months postoperatively; and then every two months thereafter. Kaplan–Meier analysis was performed to measure time to RTS and RTPIL. **Results:** In the final analysis, 65 patients were included for analysis of RTS and RTPIL, and 69 for the PROMS. In total, 81.7% (58) were male, and the mean age was 40.6 years (SD 12.5). The RTS rate was 76.9%, and the RTPIL rate was 44.6%. The mean delay to RTS was 11.6 months (SD 7.6). The median time to RTS was approximately 14 months (95% CI, 10.0–18.0). The RTPIL rate continued to increase between the first and second years after surgery. At the last follow-up, the median ATRS was 89 with an interquartile range (IQR) of [77.0–95.0], ALR-RSI was 80 [62.5–95.0], FAAM activity was 91.7 [85.7–97.6], FAAM sport was 85.9 [68.8–96.9], TAS was 7 [5–7], and UCLA was 10 [9–10]. Median follow-up was 24.8 months. **Conclusions:** In conclusion, self-reporting of RTS and RTPIL yields low rates, and caution should be used in overly optimistic preoperative counselling. Level of evidence: IV.

## 1. Introduction

Achilles tendon ruptures (ATRs) account for more than 50% of tendon ruptures related to sports activities [1]. Their reported incidence can vary greatly, with reported incidences from 6–8 per 100,000 person-years [2,3,4,5] to more than 30 per 100,000 person-years [6]. A recent meta-analysis found a pooled incidence of 15.7 per 100,000 person-years spanning from 1979 to 2021, with a global trend toward increased incidence (31.1 per 100,000 person-years in 2021) [7]. This condition is mainly related to sports, since 68–81% of ruptures are related to sports activities [3,7,8]. Men are at greater risk than women, with a relative risk of between 3 and 5.5, depending on the authors [3,7,8,9,10]. The peak incidence occurs between the ages of 30 and 49 years for males and 40 to 49 years for females, with a global trend to shift toward older age groups [3,4,7]. The pathophysiology involves tendon degeneration and often a specific traumatic mechanism, such as sudden acceleration, with plantar flexion load applied to the ankle while the knee is in extension [11,12]. Currently, the optimal management of these injuries is still debated, with surgical and conservative treatments being opposed [13]. In the athletic population, surgical treatment is frequently chosen due to the lower rate of re-rupture and the possibility of more aggressive rehabilitation [14,15].

Return to sport (RTS) is a key objective in this sports-related condition. The RTS rate reported in the literature varies greatly, ranging from 61.1 to 100% [14,16,17,18,19,20,21]. RTS is measured inconsistently across the literature. In some studies, the resumption of any sporting activity, even if different from the initial activity, is considered RTS [22]. In others, a return to a Tegner Activity Scale (TAS) equivalent to that reported before the accident defines return to sport [23], which lacks precision as it does not provide information on the possibility of resuming sporting activity at the preinjury level. This lack of consistency in the definition of RTS leads to a wide variation in the reported time to RTS, which can range from 3 to 13.4 months [21,22,24].

The aim of this study was to assess the rates of RTS and return to self-reported preinjury level (RTPIL) in the same sport among surgically treated ATR patients using direct declarative data collection. Our hypothesis was that self-reporting of sports level would yield low rates and may help prevent overly optimistic counselling.

## 2. Materials and Methods

### 2.1. Study Design and Inclusion Criteria

In this retrospective analysis of a prospective cohort, all patients treated surgically at our centre for ATR between November 2020 and September 2023 were eligible. The French Achilles Tendon Surgery Cohort Study (AchilleCDS) is listed under the Institutional Review Board (IRB) reference IRB00010835 and the ClinicalTrials.gov identifier NCT07338110. The study was approved by an ethics committee, and patients gave their informed consent. Exclusion criteria were iterative ruptures, ruptures that occurred over six weeks before surgical examination, ruptures with loss of substance requiring grafting, insertional ruptures, cases treated by transfer of the flexor hallucis longus (FHL) tendon, and patient refusal to participate. Our study design complies with the STROBE checklist.

### 2.2. Surgical Technique and Postoperative Rehabilitation Protocol

The surgical, postoperative, anesthetic, and analgesic protocols were standardized. Spinal anesthesia was administered. Surgical treatment was performed via a posteromedial approach. After opening the peritendon and debriding the tendon stumps, the tendon was sutured using a Krackow stitch with Vicryl 2 knotted in a “gift box” pattern [25] and reinforced with a peripheral overlock stitch, with the ankle at 40° plantar flexion. The peritendon and skin were then closed. Tendon length was monitored pre-, per-, and postoperatively with the Achilles Tendon Resting Angle [26].

The postoperative protocol consisted of three weeks of rest in a walking boot with heel lifts to keep the ankle in equinus position. Weight bearing was permitted in the fourth week with heel lifts to maintain equinus. These were then gradually removed over the following six weeks. The boot was finally removed after the 9th postoperative week. The rehabilitation program was standardized. Active and passive range of motion exercises were started simultaneously with weight-bearing at the fourth postoperative week. Calf-strengthening exercises were performed from the 10th week on, simultaneous with the removal of the boot. Sport clearance was allowed depending on the sport type and physiotherapist-reported progression.

### 2.3. Outcome Measures

The primary endpoint was the RTS rate, defined as the percentage of patients who answered “yes” to the question “Have you resumed your main sporting activity?” and return to self-reported preinjury level (RTPIL), defined as the percentage of patients who answered “better” or “same” to the question “If yes, your current level is?” at last follow-up. Secondary endpoints were mean delay to RTS, Patient-Reported Outcome Measures (PROMS) scores at last follow-up, Tegner Activity Scale (TAS) [27] at last follow-up, and the University of California and Los Angeles activity level rating scale (UCLA) [28,29] at last follow-up. The PROMS used in our study were the Achilles Tendon Total Rupture Score (ATRS), the Ankle Ligament Reconstruction–Return to Sport after Injury (ALR-RSI) and the Foot and Ankle Ability Measure (FAAM).

The ATRS is a self-administered questionnaire used to measure the functional outcome of patients treated for ATR. It consists of 10 questions exploring the level of physical activity and symptoms related to the condition. Responses are given on an 11-point Likert scale (between 0 and 10), allowing for a possible score between 0 and 100. The minimum clinically significant difference in score has previously been defined as between 8 and 10 points [30,31,32,33]. The French translation of the questionnaire was used (ATRS-F) [34].

The ALR-RSI is a self-administered questionnaire that assesses psychological readiness to return to sport. Developed for ankle ligament instability based on the anterior cruciate ligament (ACL)–RSI model, it has subsequently been shown to be effective for this psychological assessment in the context of ATR [35]. It consists of 12 questions constructed on 11-point Likert scales (between 0 and 10).

The FAAM is a questionnaire that separately assesses activities of daily living and sports. It includes 21 items on activities of daily living (ADL) and 8 items on sports [36]. It explores function in relation to foot and ankle pathologies and assesses the possibility of returning to sports, particularly in cases of ankle instability [37].

### 2.4. Data Collection

Data collection was performed prospectively with the AchilleCDS cohort and compiled using Websurvey^®^ software (version v59.61.0). Baseline demographics included age, gender, main sport, and preinjury sporting level (choice was given between “sedentary”, “occasional leisure”, “regular leisure”, “competition” and “professional”). A questionnaire comprising two declarative questions to assess RTS and RTPIL; ATRS, ALR-RSI, FAAM, TAS, and UCLA were sent to the patients at pre-defined follow-up times: preoperatively (postinjury); at three, six, twelve, and twenty-four months postoperatively; and then every two years. The declarative questions were “Have you resumed your main sporting activity?”; “If yes, your current level is?” with possible answers being “lower …”, “same …”, “higher than your preinjury level”; and “If no, why?” with possible answers being “changed sport” or “stopped sports”.

### 2.5. Participants and Sample Size

**Figure 1:**
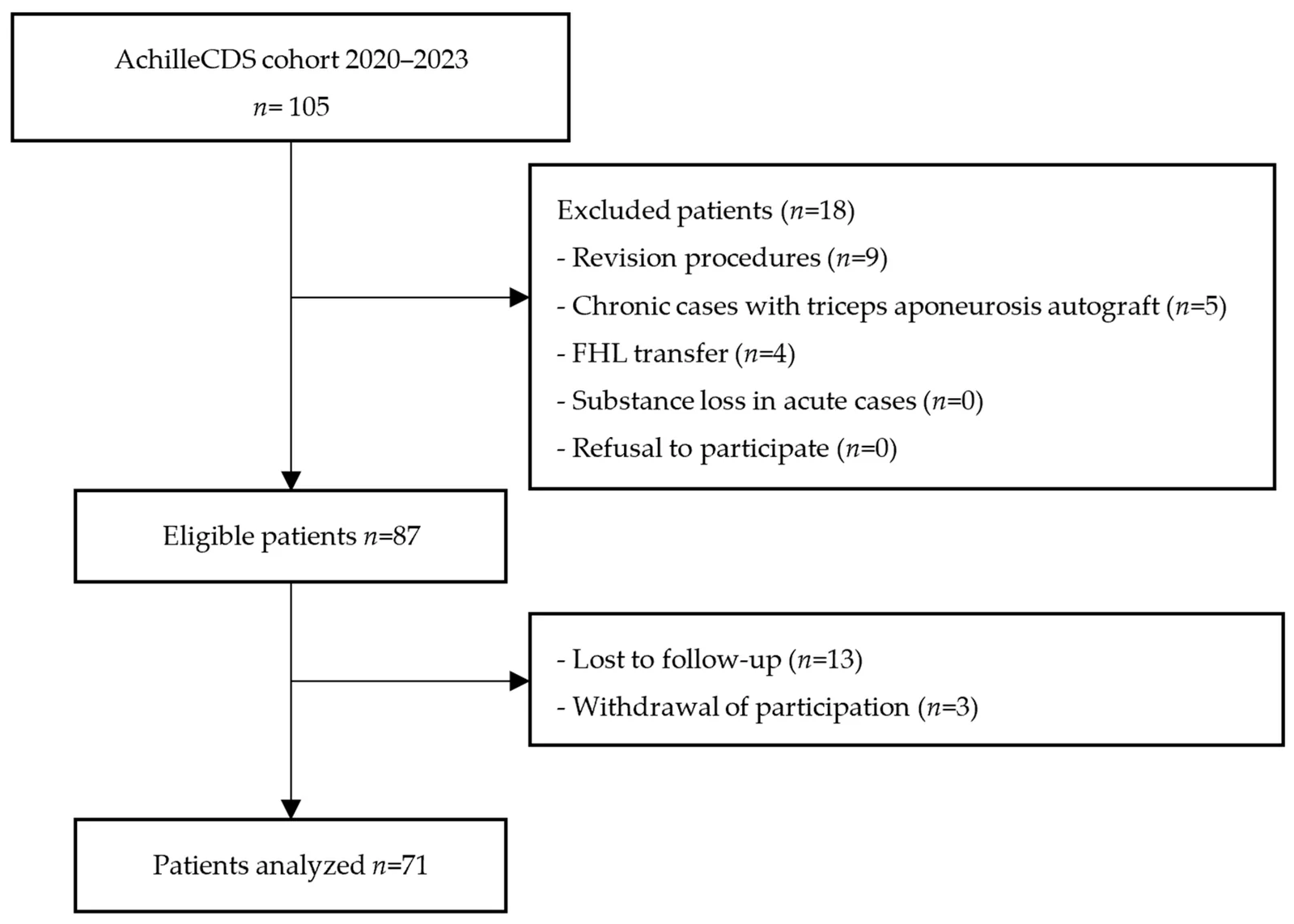
Flowchart.

Between November 2020 and September 2023, 105 patients underwent surgery for Achilles tendon rupture. Among them, 9 were revision surgeries, 5 were chronic cases (more than 6 weeks) that required triceps aponeurosis autografting, and 4 were treated with FHL transfer. We had no loss of substance in acute cases and no refusal to participate. In addition, 13 patients were lost to follow-up, and 3 withdrew from participation after inclusion and therefore did not provide any follow-up data. A total of 71 patients were therefore included in the final analysis (Figure 1). Baseline characteristics, level of sport, and type of sport practiced are summarized in Table 1.

Functional outcome scores at the last follow-up were assessed in 69 patients, excluding those with surgically treated re-ruptures. Return-to-sport analysis was performed on these 69 patients, further excluding 4 individuals who reported no sports practice prior to their injury, resulting in a total of 65 patients evaluated for this endpoint. A comparison of baseline characteristics between patients included in the final analysis (*n* = 71) and those lost to follow-up (*n* = 13) showed no significant differences between the two groups, except for the time from injury to inclusion (*p* = 0.001) (Supplementary Table S1).

### 2.6. Statistical Analysis

Continuous variables were summarized as mean and standard deviation (SD) when normally distributed and as median with interquartile range (IQR) when non-normally distributed, as assessed using the Shapiro–Wilk test. Categorical variables were presented as frequencies and percentages (N, %), and 95% confidence intervals for crude proportions were calculated using the Clopper–Pearson method. Missing data were not imputed and were excluded on a per-variable basis. Baseline characteristics were compared between included patients and those lost to follow-up using Student’s *t*-test or the Wilcoxon rank-sum test for continuous variables, depending on the normality of the distribution, and the Chi-square test or Fisher’s exact test for categorical variables, as appropriate.

Time-to-event data were analyzed using Kaplan–Meier survival analysis to estimate the time to return to sport, both at any level and at the preinjury level. The zero point on the time axis was the date of surgery. Patients who had not resumed sport at the time of the last follow-up were censored. Survival probabilities with 95% confidence intervals were derived from Kaplan–Meier estimates at pre-defined follow-up times.

A two-sided *p*-value of <0.05 was considered statistically significant.

Statistical analyses were performed using R software (version 4.6). Data management and descriptive statistics were performed using the dplyr and lubridate packages, and Kaplan–Meier survival analysis was performed using the survival and survminer packages.

## 3. Results

The RTS rate at last follow-up was 76.9% (50/65, 95% CI: 64.8–86.5). The RTPIL rate was 44,6% (29/65, 95% CI: 32.3–57.5) (Table 2). The mean delay to RTS among patients who returned to sport was 11.6 months (SD 7.6 months). At the last follow-up, the median ATRS was 89 [IQR: 77.0–95.0], the median ALR-RSI was 80 [IQR: 62.5–95.0], the median FAAM activity was 91.7 [IQR: 85.7–97.6], the median FAAM sport was 85.9 [IQR: 68.8–96.9], the median TAS was 7 [IQR: 5–7] and the median UCLA was 10 [IQR: 9–10] (Table 3). There were three postoperative complications: two re-ruptures (2.8%) and one infection (1.4%). Median follow-up was 24.8 months.

**Figure 2:**
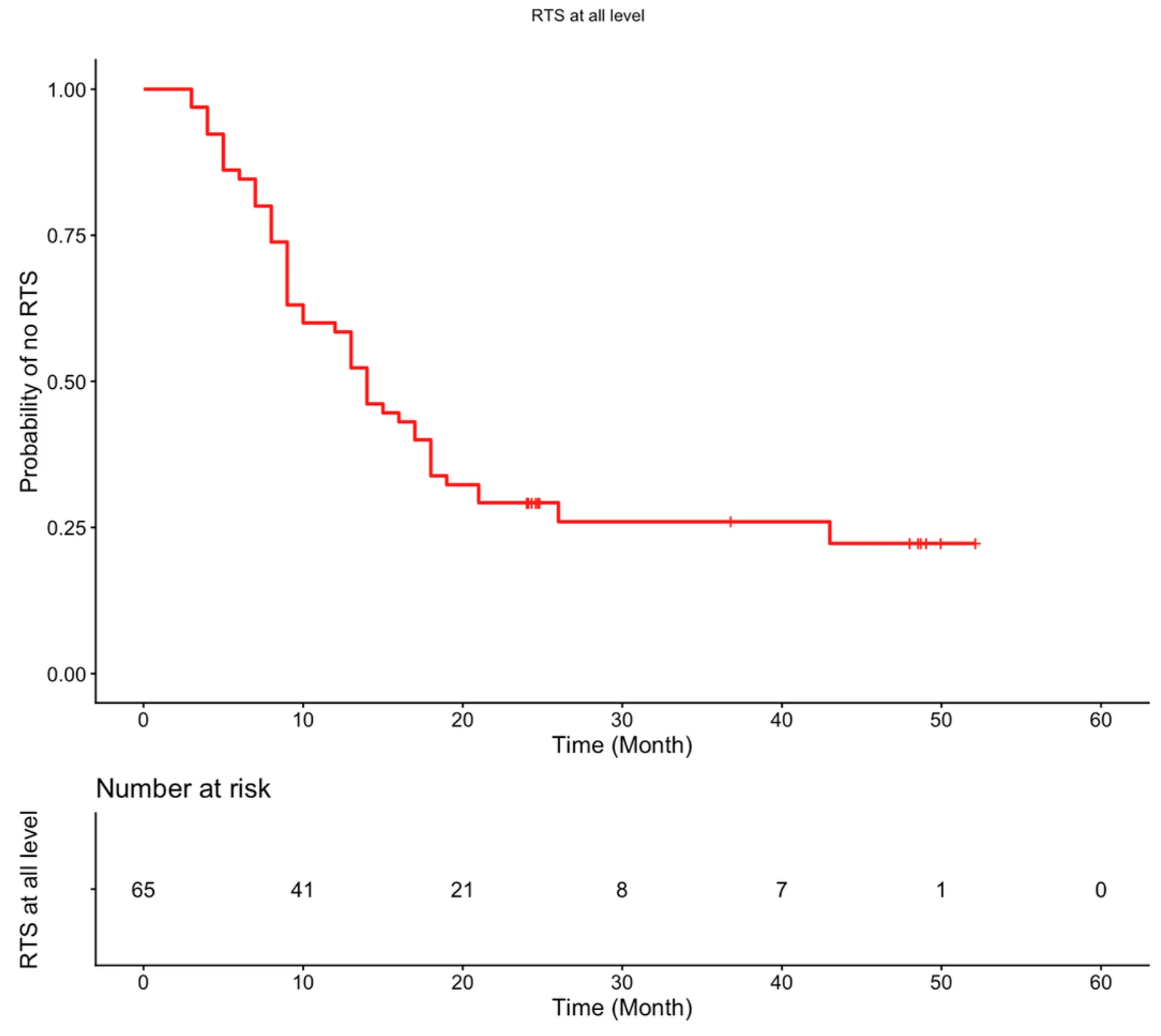
Kaplan–Meier curve showing time to return to sport at any level after Achilles tendon surgery.

For RTS at any level, 17.5% of patients had resumed some level of sport at 6 months, increasing to 43.9% at 12 months, 70.2% at 24 months, and 78.7% at 48 months. The median time to RTS was approximately 14 months (95% CI, 10.0–18.0). The probability of not yet having returned to sport was 0.82 (95% CI, 0.73–0.93) at 6 months and 0.21 (95% CI, 0.12–0.39) at 48 months (Figure 2).

**Figure 3:**
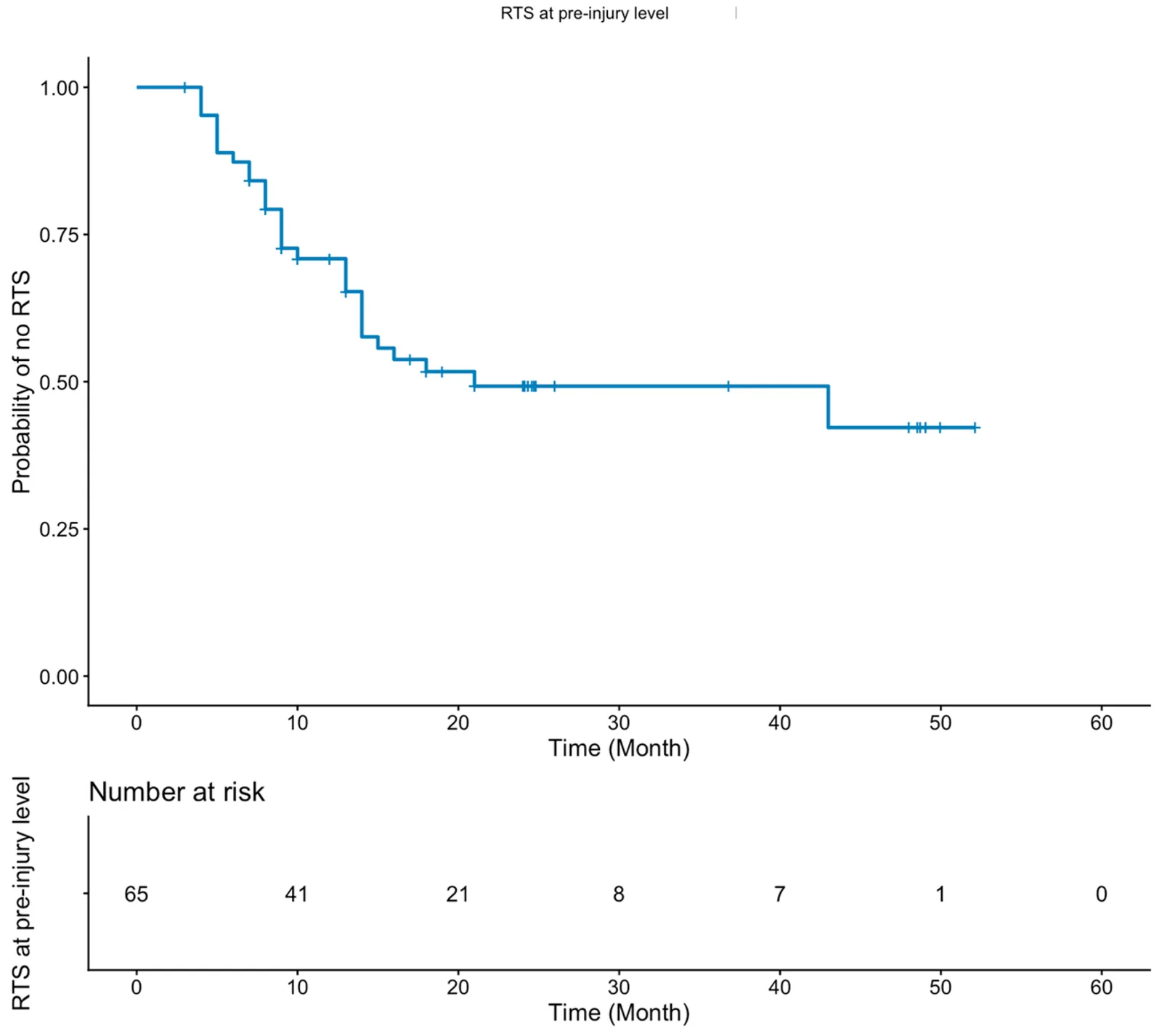
Kaplan–Meier curve showing time to return to sport at the same preinjury level after Achilles tendon surgery.

Kaplan–Meier analysis showed a gradual increase in the proportion of patients returning to their preinjury status over time. At 6 months postoperatively, 14.5% of patients had resumed sport at the same level, increasing to 29.7% at 12 months, 48.1% at 24 months, and 56.8% at 48 months. This estimate differs from the raw RTPIL rate (44.6%, Table 2) due to censoring of patients with shorter follow-up. The median time to RTPIL could not be estimated, as fewer than 50% of patients had reached this outcome during follow-up. The estimated probability of not yet returning to the same level was 0.85 (95% CI, 0.77–0.95) at 6 months and 0.43 (95% CI, 0.28–0.68) at 48 months (Figure 3).

## 4. Discussion

In our study, the RTS was 76.9% and the RTPIL was 44,6%. In a meta-analysis of 108 studies including 6506 patients, the overall RTS rate was 80%, and the authors described how studies that defined RTS in their methodology had a 77% RTS rate whereas those who did not had an RTS rate of 91% [21]. Moreover, a recent systematic review of 9 studies, including 748 patients, 84% being male, found a 77.4 RTS rate [38]. Our data demonstrated that self-reported RTS and RTPIL yield different proportions, suggesting that the definition of RTS influences its outcome. The low RTPIL rate shows that perceived performance is often altered after ATR. Our measurement is based on a declarative, simple question. A patient may report returning to the “same level” while still having reduced training volume, fewer matches or sessions, avoidance of sprinting, jumping, or rapid changes of direction, persistent calf-strength deficit, reduced heel-rise height or endurance, or a change from competitive participation to recreational participation within a broadly similar activity category. Conversely, a patient may have objectively good strength and function but not return because of fear of reinjury, reduced motivation, family obligations, or loss of sporting opportunity. Psychological readiness and fear of movement can materially affect return after Achilles tendon rupture.

RTS is approached differently depending on whether the population is professional athletes or not. In a population of professional athletes, RTS is often defined as a return to play in the same league as before the ATR. RTS rate therefore varies mainly according to the type of sport practiced and the specific constraints of these sports on the Achilles tendon. RTS rate for NFL players has been reported to be between 65.6 and 72.5% [18], but varies according to the position within the team. So-called “skilled” positions such as linebackers and runningbacks have RTS rates as low as 52.8% [39]. Conversely, the RTS rate for soccer players can reach 94.9% [18]. This seems to indicate that the specificities of each sport concerning contact, accelerations and decelerations influence RTS.

In the non-professional population, the RTS rate varies greatly depending on how it is defined in studies. Bak et al. chose to analyze RTS as a return to any sporting activity, at any level [22]. Lerch et al. defined RTS as a TAS at follow-up equivalent to the pre-accident TAS [23]. We do not believe that this latter definition is sufficiently precise to meet the functional needs of patients, who will subjectively feel the difference compared to their pre-accident level. Generally, the definition of RTS should be standardized to allow comparison.

The survival analysis in our study highlights the dynamics of resuming sporting activities after ATR. The RTS and RTPIL rates gradually increased during the first two years after surgery. At 24 months, more than 70% of our patients had resumed their sporting activity, but less than half had returned to their preinjury level. Olsson et al. showed that functional scores and physical tests changed little between the first and second postoperative years [40]. Conversely, in our study, the RTS and RTPIL rates increased between 12 and 24 months. However, in the short term, relatively few patients reported being able to resume their activities. At 12 months, only 43.9% had been able to resume their sporting activities. This may be related to the type of sport practiced by our patients, 50 (70%) of whom reported practicing a pivot or pivot contact sport. In a study about the ability of the Ankle-GO score, a composite score based on four functional tests and two PROMS, to assess the RTS in surgically treated ATR patients, Lopes et al. reported a 28% RTPIL rate at 9 months [41]. In this study, the Ankle-GO score was able to predict RTS at 6 months, which is a useful tool in the follow-up of ATR patients.

In the study by Lerch et al. analyzing return to sport in the general population after conservative treatment and accelerated rehabilitation, the main risk factor identified was the level of preinjury sporting activity, as measured on the Tegner Activity Score (TAS) scale. Patients with low activity (TAS < 6) returned significantly less (67% vs. 91%) than those with high activity (TAS ≥ 6) [23]. Conversely, in a literature review of surgically treated patients, the lowest rate of return to sport (61%) was found in a study of a non-athletic population [24]. We did not design this study to have enough power to assess risk factors for RTS. Further research will be needed to help determine how sport type and preinjury activity level affect RTS after ATR.

The functional scores in our study were satisfactory at last follow-up. The median ATRS at the last follow-up was 89, which is similar to the results published following surgical treatment [42,43,44]. The median ALR-RSI was 80 at the last follow-up. Shitrit et al. previously showed that high ALR-RSI scores were associated with RTS [35]. At the last follow-up, the FAAM activity score was 91.7 on average. The minimum clinically important difference for the FAAM is 8 and 9 points for the activity and sport subcategories, respectively [36]. This corresponds to the within-person change beyond which patients report to have improved (in activities of daily living or sports) after 4 weeks of physiotherapy. At the last follow-up, the patients in our study therefore reported minimal limitations in activities of daily living. The median FAAM sport score was 85.9, indicating good self-reported ability to participate in sports.

In our study, the re-rupture rate was 2.8%, and the infection rate was 1.4%, which is consistent with the literature [45,46]. There were no recorded cases of deep vein thrombosis, which may be explained by a lack of detection of this complication, as it is often screened for and treated outside our centre.

This study has some limitations: it is a small single-centre cohort with a retrospective analysis. It does not have a comparative group in its design. The sample is predominantly male and mixes sporting levels. Declarative collection of data is associated with declarative bias and there is a possible recall error for preinjury sport level. The 48-month Kaplan–Meier estimates should be interpreted with caution given the small number of patients at risk at this time point. Censored patients may be heterogeneous, and the non-informative censoring assumption could not be formally verified. Of 87 eligible patients, 16 (18%) were lost to follow-up or withdrew participation after inclusion, which is not negligible. Patients lost to follow-up showed no statistical difference in baseline characteristics compared to the patients included in the final analysis (Supplementary Table S1) except for time from injury to inclusion. Patients lost to follow-up had a longer time from injury to inclusion (median 4.6 vs. 0.3 months), possibly reflecting lower engagement with care that may extend to follow-up participation and RTS itself. Two patients were excluded from the final analysis for having a re-rupture needing surgical treatment. While it is common to exclude patients presenting this adverse event, it is still clinically relevant, and those patients may not achieve RTS or RTPIL. The RTS and RTPIL rate of treated re-ruptured should however be specifically investigated. We did no objective strength/performance testing. The follow-up is heterogeneous, and there is attrition. Additionally, data on smoking status, diabetes, prior tendinopathy, and exposure to fluoroquinolones or glucocorticosteroids—all potential confounders of RTS—were not systematically recorded in our cohort and could therefore not be included in the analysis. Finally, there was no prior calculation of statistical power.

Our study also has some strengths. The collection of RTS data on a declarative basis allows us to integrate the psychological component related to athletic performance and to assess whether the patient feels any functional sequelae from their ATR injury. The collection of PROMS further complements this aspect.

## 5. Conclusions

The RTS rate at the last follow-up after surgical treatment of an ATR injury was 76.9% in our study. RTPIL rate was 44.6%, and the mean delay to RTS was 11.6 months (SD 7.6 months). The median time to RTS was approximately 14 months (95% CI, 10.0–18.0). RTS occurs gradually during the first two years after surgery. Our data demonstrated that self-reported RTS and RTPIL yield different proportions, suggesting that the definition of RTS influences its outcome. Self-reported performance is strongly altered by ATR.

## Figures and Tables

**Table 1:** Population characteristics and preoperative/preinjury PROMS.

	Overall
	*n* = 71
**Sex**	
Female	13 (18.3%)
Male	58 (81.7%)
**Age, mean (sd)**	40.6 (12.5)
**BMI, mean (sd)**	26.2 (3.0)
**Side**	
Right	38 (53.5%)
Left	33 (46.5%)
**Main sport**	
No sport	4 (5.6%)
Linear sport (Running, cycling)	17 (23.9%)
Pivot sport (skiing, tennis, squash, badminton, volleyball, dance, gymnastics, golf)	18 (25.4%)
Pivot contact sport (Football/soccer, rugby, basketball, handball, hockey, combat sports)	32 (45.1%)
**Sport level**	
Sedentary	4 (5.6%)
Professional	2 (2.8%)
Competition	19 (26.8%)
Regular leisure	40 (56.3%)
Occasional leisure (active)	6 (8.5%)
Time between injury and surgery in months, median (IQR)	0.3 (0.1, 0.7)
Preoperative ALR-RSI score, median (IQR)	53.3 (35.8–87.5)
Preoperative FAAM Sports score, median (IQR)	32.8 (0.0–90.6)
Preoperative FAAM Activities score, median (IQR)	70.8 (8.8–97.6)
UCLA score before injury, median (IQR)	10.0 (9.0–10.0)
TEGNER score before injury, median (IQR)	7.0 (5.0–7.0)
Preoperative intensity of the pain, median (IQR)	3.0 (1.0–7.0)
Follow-up duration in months, median (IQR)	24.8 (24.1–48.3)

**Table 2:** Return to sport and self-reported preinjury level rate.

Return to Sport	*n* = 65
No	15 (23%)
Yes	50 (77%)
If yes,	
**Time to return in months, mean (sd)**	11.6 (7.6)
**Level of return**	
Lower	21 (42%)
Same	22 (44%)
Higher	7 (14%)

**Table 3:** Patient-reported outcome measures.

	*n* = 69
FAAM Activities score, median (IQR)	91.7 (85.7–97.6)
FAAM Sports score, median (IQR)	85.9 (68.8–96.9)
ALR-RSI score, median (IQR)	80.0 (62.5–95.0)
ATRS-F score, median (IQR)	89.0 (77.0–95.0)
TEGNER score, median (IQR)	7 (5.00–7.00)
UCLA score, median (IQR)	10 (9.00–10.00)
Intensity of the pain, median (IQR)	0.5 (0.0–2.0)
Status compared to preoperative	
Same	15 (22%)
Slightly better	6 (9%)
Slightly worse	19 (28%)
Better or much better	24 (35%)
Worse	3 (4%)
Unknown	2 (3%)

## Data Availability

Data is available upon reasonable request.

## Supplementary Materials

The following supporting information can be downloaded at https://www.mdpi.com/article/10.3390/jcm15176512/s1. Supplementary Table S1: Comparison of Lost to Follow Up to Included Patients. Supplementary Table S2: STROBE check list and statement.

## Abbreviations

The following abbreviations are used in this manuscript:

RTS	Return To Sport
RTPIL	Return To Preinjury Level
PROMS	Patient-Reported Outcome Measures
ATRS	Achilles Tendon Total Rupture Score
ALR-RSI	Ankle Ligament Reconstruction–Return to Sport after Injury
FAAM	Foot and Ankle Ability Measurement
TAS	Tegner Activity Score
UCLA	University of California and Los Angeles
ATR	Achilles Tendon Rupture
IRB	Institutional Review Board
FHL	Flexor Hallucis Longus
ADL	Activity of Daily Living
SD	Standard Deviation
IQR	Interquartile Range
