# Supplementary material for: Return to Sport and Return to Self-Reported Preinjury Level After Surgical Repair of Acute Achilles Tendon Rupture: A 2-Year Prospective Cohort Analysis
Source: J Clin Med. 2026 Aug 23;15(17):6512. doi: 10.3390/jcm15176512 (PMC13566404; doi:10.3390/jcm15176512)
Supplement: Supplementary file 1 [file jcm-15-06512-s001.zip › jcm-4492019-supplementary.pdf]

Supplementary Table S1: comparison of lost to follow up included patients
Supplementary Table S2: STROBE check list and statement

Characteristic	Included (N = 71)	Lost to follow-up (N = 13)	*p*-value
**Sex, n (%)**			0.20
Female	13 (18.3%)	0 (0.0%)	
Male	58 (81.7%)	13 (100.0%)	
**Age, years**	40.6 (12.5)	50.9 (18.8)	0.071
**BMI, kg/m²**	26.0 (3.1)	27.1 (1.7)	0.11
**Side, n (%)**			0.070
Right	37 (52.9%)	3 (23.1%)	
Left	33 (47.1%)	10 (76.9%)	
**Primary sport, n (%)**			0.80
No sport	4 (5.6%)	1 (7.7%)	
Non-pivot sport	17 (23.9%)	4 (30.8%)	
Pivot sport	18 (25.4%)	4 (30.8%)	
Pivot/contact sport	32 (45.1%)	4 (30.8%)	
**Sport level, n (%)**			0.60
Competitive	19 (26.8%)	4 (30.8%)	
Occasional recreational (active)	8 (11.3%)	3 (23.1%)	
Regular recreational	41 (57.7%)	5 (38.5%)	
Professional	2 (2.8%)	0 (0.0%)	
Sedentary	1 (1.4%)	1 (7.7%)	
**Time from injury, months**	0.3 (0.1–0.7)	4.6 (0.7–11.9)	0.001
Preoperative ALR-RSI score	53.3 (35.8–87.5)	58.3 (27.5–80.0)	0.90
Preoperative FAAM Sports score	32.8 (0.0–90.6)	21.9 (6.3–78.1)	>0.90
Preoperative FAAM Activities of Daily Living score	70.8 (8.8–97.6)	65.5 (40.5–91.7)	>0.90
Pre-injury UCLA Activity Scale	10.0 (9.0–10.0)	10.0 (7.5–10.0)	0.90
Pre-injury Tegner Activity Scale	7.0 (5.0–7.0)	6.0 (4.0–8.5)	0.60
Preoperative ankle pain intensity (0–10)	3.0 (1.0–7.0)	2.0 (0.0–6.0)	0.30

STROBE Statement—Checklist of items that should be included in reports of ***cohort studies***

We hereby certify that we checked our manuscript to be in compliance with the STROBE check list.

	Item No	Recommendation
**Title and abstract**	1	(*a*) Indicate the study's design with a commonly used term in the title or the abstract
		(*b*) Provide in the abstract an informative and balanced summary of what was done and what was found
**Introduction**		
Background/rationale	2	Explain the scientific background and rationale for the investigation being reported
Objectives	3	State specific objectives, including any prespecified hypotheses
**Methods**		
Study design	4	Present key elements of study design early in the paper
Setting	5	Describe the setting, locations, and relevant dates, including periods of recruitment, exposure, follow-up, and data collection
Participants	6	(*a*) Give the eligibility criteria, and the sources and methods of selection of participants. Describe methods of follow-up
		(*b*) For matched studies, give matching criteria and number of exposed and unexposed
Variables	7	Clearly define all outcomes, exposures, predictors, potential confounders, and effect modifiers. Give diagnostic criteria, if applicable
Data sources/ measurement	8*	For each variable of interest, give sources of data and details of methods of assessment (measurement). Describe comparability of assessment methods if there is more than one group
Bias	9	Describe any efforts to address potential sources of bias
Study size	10	Explain how the study size was arrived at
Quantitative variables	11	Explain how quantitative variables were handled in the analyses. If applicable, describe which groupings were chosen and why
Statistical methods	12	(*a*) Describe all statistical methods, including those used to control for confounding
		(*b*) Describe any methods used to examine subgroups and interactions
		(*c*) Explain how missing data were addressed
		(*d*) If applicable, explain how loss to follow-up was addressed
		(*e*) Describe any sensitivity analyses
**Results**		
Participants	13*	(a) Report numbers of individuals at each stage of study—eg numbers potentially eligible, examined for eligibility, confirmed eligible, included in the study, completing follow-up, and analysed
		(b) Give reasons for non-participation at each stage
		(c) Consider use of a flow diagram
Descriptive data	14*	(a) Give characteristics of study participants (eg demographic, clinical, social) and information on exposures and potential confounders
		(b) Indicate number of participants with missing data for each variable of interest
		(c) Summarise follow-up time (eg, average and total amount)
Outcome data	15*	Report numbers of outcome events or summary measures over time

Main results	16	(*a*) Give unadjusted estimates and, if applicable, confounder-adjusted estimates and their precision (eg, 95% confidence interval). Make clear which confounders were adjusted for and why they were included
		(*b*) Report category boundaries when continuous variables were categorized
		(*c*) If relevant, consider translating estimates of relative risk into absolute risk for a meaningful time period
Other analyses	17	Report other analyses done—eg analyses of subgroups and interactions, and sensitivity analyses
**Discussion**		
Key results	18	Summarise key results with reference to study objectives
Limitations	19	Discuss limitations of the study, taking into account sources of potential bias or imprecision. Discuss both direction and magnitude of any potential bias
Interpretation	20	Give a cautious overall interpretation of results considering objectives, limitations, multiplicity of analyses, results from similar studies, and other relevant evidence
Generalisability	21	Discuss the generalisability (external validity) of the study results
**Other information**		
Funding	22	Give the source of funding and the role of the funders for the present study and, if applicable, for the original study on which the present article is based

*Give information separately for exposed and unexposed groups.
